# Mining of the CULLIN E3 ubiquitin ligase genes in the whole genome of *Salvia miltiorrhiza*

**DOI:** 10.1016/j.crfs.2022.10.011

**Published:** 2022-10-08

**Authors:** Xiankui Gao, Xiujuan Li, Chengan Chen, Can Wang, Yuqi Fu, ZiZhen Zheng, Min Shi, Xiaolong Hao, Limei Zhao, Minghua Qiu, Guoyin Kai, Wei Zhou

**Affiliations:** a Laboratory for Core Technology of TCM Quality Improvement and Transformation, School of Pharmaceutical Sciences, The First Affiliated Hospital, Zhejiang Chinese Medical University, Hangzhou, Zhejiang, 311402, PR China; b State Key Laboratory of Phytochemistry and Plant Resources in West China, Kunming Institute of Botany, Chinese Academy of Sciences, Kunming, 650201, PR China

**Keywords:** *Salvia miltiorrhiza*, Gene family, Expression pattern, Phenolic acid, Tanshinone, CULLIN E3 ubiquitin ligase

## Abstract

CULLIN (CUL) proteins are E3 ubiquitin ligases that are involved in a wide variety of biological processes as well as in response to stress in plants. In *Salvia miltiorrhiza*, *CUL* genes have not been characterized and its role in plant development, stress response and secondary metabolite synthesis have not been studied. In this study, genome-wide analyses were performed to identify and to predict the structure and function of *CUL* of *S. miltiorrhiza*. Eight *CUL* genes were identified from the genome of *S. miltiorrhiza*. The *CUL* genes were clustered into four subgroups according to phylogenetic relationships. The CUL domain was highly conserved across the family of *CUL* genes. Analysis of *cis*-acting elements suggested that *CUL* genes might play important roles in a variety of biological processes, including abscission reaction acid (ABA) processing. To investigate this hypothesis, we treated hairy roots of *S. miltiorrhiza* with ABA. The expression of *CUL* genes varied obviously after ABA treatment. Co-expression network results indicated that three *CUL* genes might be involved in the biosynthesis of phenolic acid or tanshinone. In summary, the mining of the *CUL* genes in the whole genome of *S. miltiorrhiza* contribute novel information to the understanding of the *CUL* genes and its functional roles in plant secondary metabolites, growth and development.

## Highlights

• Eight CUL ubiquitin ligase genes were identified from the genome of *Salvia miltiorrhiza.*
• The *CUL* genes were clustered into four subgroups according to phylogenetic relationships.
• The expression of seven *CUL* genes varied obviously after ABA treatment.
• Three *CUL* genes might be involved in the phenolic acid or tanshinone biosynthesis.

## Graphical abstract

**Figure:**
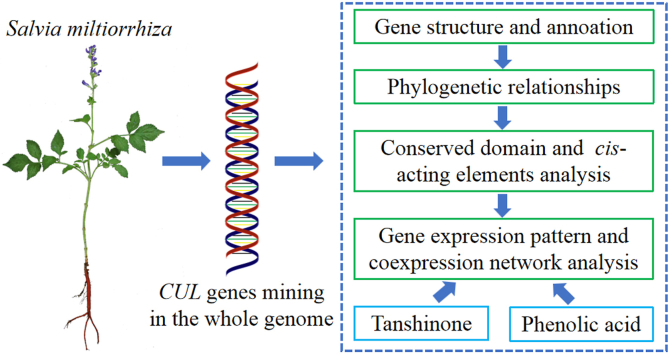

## 1 Introduction

*Salvia miltiorrhiza* is a famous Chinese medicinal plant used in medicine and health food for thousands of years (Qian et al., 2022). It has been used to treat cardiovascular and cerebrovascular diseases in many countries. So far, the *S. miltiorrhiza* has become a model of Chinese herbal medicine due to its characteristics of being widely and deeply studied (Shi et al., 2016; Huang et al., 2021; Zhou et al., 2021a, b; Sun et al., 2022). The active ingredients of *S. miltiorrhiza* include two groups: one group is diterpenoid tanshinone, including tanshinone I, tanshinone IIA, tanshinone IIB, dihydrotanshnone I and cryptotanshinone, exhibits various pharmacological activities including antioxidant, antitumor and anti-inflammatory properties; the other group is water-soluble phenolic acids, such as rosmarinic acids, salvianolic acids and lithospermic acid, functions as antibacterial, anti-oxidative and antiviral reagents. (Sun et al., 2022; Zhao et al., 2022). These components have been shown to exhibit various biological activities, including anti-tumor, anti-inflammatory, and antibacterial effects (Liu et al., 2022; Sun et al., 2022; Zhao et al., 2022). During their life courses, plants are repeatedly exposed to various abiotic stresses such as drought, salt, and low temperatures, resulting in oxidative damage and adverse effects (Gupta et al., 2020; Smokvarska et al., 2020; Wang et al., 2021). Plants have evolved complex, efficient mechanisms to cope with unfavorable environment. The response of transcriptional regulation, post-transcriptional modification, epigenetic regulation, and secondary metabolism to abiotic stress has been studied in previous studies in *S. miltiorrhiza* (Marino et al., 2013; Dou et al., 2021; Karre et al., 2021; Tong et al., 2021; Wang et al., 2021). But, ubiquitination modification and degradation of functional proteins regulating the synthesis of medicinal active substance in *S. miltiorrhiza* is still unclear.

Ubiquitination is a crucial post-translational modification (Chen et al., 2021; Wang et al., 2021). The ubiquitin/26S proteasome system (UPS) is a pervasive and effective route for protein removal in eukaryotes. UPS include ubiquitin (Ub), ubiquitin-activating enzyme (E1), ubiquitin-conjugating enzyme (E2), ubiquitin-ligating enzyme (E3), and the 26S proteasome (Trujillo and Shirasu 2010; Chen et al., 2021). Ub is bound to specific proteins and functions in target proteins’ degradation by the E1–E2–E3 multi-enzyme cascade, while E3 are thought to be the key factor to define substrate specificity during the process of ubiquitination and degradation (Richburg et al., 2014; Serrano et al., 2018). E3 were classified into four main types as U-box, HECT (Homology to E6-Associated Carboxy-Terminus), RING (Really Interesting New Gene) and Cullin–RING ligases (CRLs) through their reaction mechanism and subunit compositions (Vierstra 2009). CUL proteins are molecular scaffolds and play a crucial role in ubiquitin-mediated post-translational modification of cellular proteins. CUL proteins are also present in model organisms like *Drosophila melanogaster*, *Caenorhabditis elegans*, *Arabidopsis thaliana* and *yeast* (Chen et al., 2009; Sarikas et al., 2011; Ban and Estelle 2021).

CUL proteins possess a substrate-targeting function, often through an adaptor protein and a RING finger component (Sarikas et al., 2011; Liu et al., 2017). All the complexes known so far have been grouped into four main CRLs. The classes are consist of: 1) the CUL/RING/Skp/F-box CRLs proteins acting as substrate receptors while Skp1 or related proteins serving as adaptors; 2) the CUL/RING/BTB CRLs (BTB CRLs) protein being characterized by the lack of additional adaptors and containing proteins with BTB domains as substrate receptors directly attach to CULs (Christians et al. 2009, 2012; Marin 2009); 3) the CUL/RING/DDB/DCAFs CRLs (DDB CRLs) protein related to mammalian DAMAGE-SPECIFIC DNA-BINDING PROTEIN 1 protein (DDB1) serving as adaptors and with WD40 domains acting as substrate receptors (Marin 2009); 4) the complex receptors (BC-box CRLs) consisting of CUL/RING/Elongin/SOCSB boxes as substrate receptors and containing elongin proteins as adaptors (Marin 2009; Chahtane et al., 2018; Julian et al., 2019; Chico et al., 2020). The CUL-organized CRLs recruits the substrate and the E2 ubiquitin-conjugating enzymes, which transfer ubiquitin from the E2-conjugating enzymes to the substrate. In addition, conjugation of CULs with the ubiquitin-like molecule Nedd8 modulates activation of the corresponding CRL complex through conformational regulation of the interactions between CUL's carboxyterminal tail and CRL's RING subunit. In plants, CRLs are probably the best-characterized E3s to date, participating in plant growth and development (Roberts et al., 2011; Chen et al., 2013; Genschik et al., 2013; Chahtane et al., 2018).

The dissection of the whole genome of *S. miltiorrhiza* provides an excellent molecular biology platform for its gene family analysis, functional gene mining, genome evolution, and so on (Schwechheimer 2018). So far, the *CUL* gene family of *S. miltiorrhiza* is rarely studied. *CUL* genes were thought to play vital roles in regulating the growth and development of *S. miltiorrhiza*, therefore, it is essential to investigate the *CUL* gene family in *S. miltiorrhiza*. The present study systematically studied the *CUL* genes number, gene structures, conserved domains and subgroup classification in the whole genome of *S. miltiorrhiza*. Moreover, we investigated gene expression profiles in different tissues along with the ABA treatment, providing a valuable reference for the functional identification of *CUL* genes.

## 2 Materials and methods

### 2.1 Sequence retrieval and characterization

To identify the potential *CUL* E3 in *S. miltiorrhiza*, the genome sequence was downloaded from the *S.miltiorrhiza* database (Xu et al., 2016) (ftp://danshen.ndctcm.org:10402/). Then, the seed file of the CULLIN domain (PF00888) was retrieved from the Pfam database. The HMMER program was used to identify the potential *CUL* genes in *S. miltiorrhiza* (Eddy 2011; Finn et al., 2011). All candidate *CUL* genes obtained from the result of HMMsearch were further submitted to SMART website (http://smart.embl-heidelberg.de/) to determine completeness of CUL conserved domain (Letunic and Bork 2018; Wang et al., 2021). In addition, ExPASy-Compute pI/Mw tool was used to calculate the amino acid number, molecular weight, theoretical pI, instability index, and aliphatic index as well as GRAVY (Grand Average of Hydropathicity) (Chen et al., 2022). A Cell PLoc 2.0 prediction was introduced to determine the subcellular localization of *CUL* gene candidates (http://www.csbio.sjtu.edu.cn/bioinf/plant-multi/) (Emanuelsson et al., 2000; Chen et al., 2022).

### 2.2 Phylogenetic tree construction

CUL protein sequences from *A. thaliana* and *S. miltiorrhiza* were collected. In final, the total of 14 CUL protein sequences were downloaded. Sequence alignment was performed using MEGA 6.0 software with the ClustalW function (Tamura et al., 2013). The phylogenetic tree was constructed by MEGA 6.0 with the Neighbor-joining method. The ITOL tool was used to create the tree visualization (https://itol.embl.de/) (Letunic and Bork 2019; Sharma and Taganna 2020; Sharma et al., 2021).

### 2.3 Identification of gene structure and protein motif

To identify and visualize the structural organization (introns, exons and untranslated regions) of the *S. miltiorrhiza CUL* genes, the GSDS (Gene Structure display System) tool was utilized (http://gsds.cbi.pku.edu.cn/) (Hu et al., 2015). The novel reserved motifs of *S. miltiorrhiza CUL* genes were identified using a motif-based sequence analysis tool by MEME suite (http://meme-suite.org/) (Bailey et al., 2009; Sharma and Taganna 2020; Sharma et al., 2021). For a total of three motifs and a width limit of 50 amino acids were used in this study. Meanwhile, the *CUL* genes structures and conserved domains are visualized using the Tbtools software (Chen et al., 2020a; Du et al., 2022).

### 2.4 Promoter analysis

A length of 3000-bp in the upstream of initiation codon of the *CUL* genes was specified as putative promoter sequences. Eight promoter sequences of *CUL* genes were retrieved using Tbtools (Chen et al., 2020a). The *cis*-acting regulatory elements of the promoter sequences were predicted by PlantCare online (Rombauts et al., 1999). According to the functional annotations of *cis*-acting elements, the candidate elements were gathered for further research and the *cis*-acting elements with the same functional annotations were incorporated into the same group. Additionally, the word art image of *cis*-acting elements in the promoters was generated with the WordArt tool (https://wordart.com) (Mi et al., 2005; Sharma and Taganna 2020).

### 2.5 Hairy root treated with ABA, Illumina sequencing and De novo transcriptome assembly

Sterile *S. miltiorrhiza* plants were cultivated on Murashige and Skoog (MS) media at 25 °C under a 16 h light/8 h dark photoperiod (Deng et al., 2020b; Zhou et al., 2021b). The *Agrobacterium rhizogenes* strain C58C1 (pRi A4) cultures were used to infect the sterile stems and/or leaves of *S. miltiorrhiza* to generate hairy roots (Cao et al., 2018; Huang et al., 2019). The well-grown *S.miltiorrhiza* hairy roots were used to perform the different treatment with ABA, and the hairy roots were collected after 0h, 0.5h, 1h, 2h, 4h and 8h of treatment for RNA isolation and cDNA synthesis (Du et al., 2018; Cao et al., 2021; Zheng et al., 2021).

Reverse transcription was performed with the cDNA Synthesis Kit (Clontech, USA) according to protocols. Double stranded cDNAs were separated on agarose gel, and were recovered for the RNA-seq (Chen et al., 2022). Construction of the cDNA library was performed by the Majorbio Bio-pharm Technology (Shanghai, China) and was sequenced by Illumina HiSeq TM 2500 with PE100. All reads have been uploaded to the National Center for Biotechnology Information (NCBI) public database with the SRA access number SRP307198. *De novo* assembly of the Illumina sequenced short length reads was conducted as reported previously (Zhou et al., 2017). Using the RNA-seq data, the expression levels of these genes were quantified by RPKM values, and TBtools visualized the expression results (Chen et al., 2020a). Based on the FPKM values of genes in transcriptome database, the co-expression regulatory network of tanshinone and salvianolic acid biosynthetic genes with candidate *CUL* genes was constructed by Pearson's correlation test (r > 0.8 and *P*-value < 0.05). Then, Cytoscape software is used to visualize the above results (Shannon et al., 2003).

### 2.6 Gene expression profiles detected by quantitative real-time PCR (qRT-PCR)

Different tissues (roots, stems, leaves, and flowers) and hairy roots grown for 40 days were treated with 50 μM ABA for RNA isolation, and was converted into cDNA using a plant RNA prep pure kit (Tiangen Biotech Co., Ltd., Beijing, China) (Zhou et al., 2021a). cDNA of each sample was synthesized and qRT-PCR assay was carried out using a Super Real PreMix Plus (SYBR Green) kit (Tiangen, China) on ABI Step One Plus Real-TimePCR Systems (Applied Biosystems, USA) as described previously. *SmActin* gene was used as an internal control. The primer pairs for qRT-PCR are summarized in Table S1. The 2^−ΔΔCt^ method was introduced to perform the quantification of gene expression (Liu et al., 2022). Each generated data point represented the average of three independent experiments.

## 3 Result

### 3.1 Identification and characterization of CUL gene family

In this study, we used a strict pipeline to identify *CUL* genes in the *S. miltiorrhiza* genome. The HMM (Hidden Markov Model) profile of the CUL domain was obtained from the Pfam database. The HMMER tools were employed to convert the multiple sequence alignment into the position-specific scoring system, then to conduct large-scale sequence analysis. In final, we obtained eight putative sequences using HMMER with default parameters and a significant *P*-value of 0.01. We have analyzed the molecular weight, number of amino acids, gene length, pI, instability index, grand average of hydropathy, introns, class, and subcellular localization of all the *CUL* genes (Table S2). The molecular weight of the *CUL* genes ranges from 32.55 to 103 kDa, and the number of amino acids varies from 280 to 920. The pI value is from 4.98 to 8.40. Most of the proteins were predicted to be unstable and hydrophilic. From the protein subcellular localization, it was predicted that most of the CUL proteins might locate in the cytoplasm, while the remaining members were predicted to exist in the chloroplast **(**Table S2**)**.

### 3.2 Phylogenetic relationships of CUL genes

**Fig. 1:**
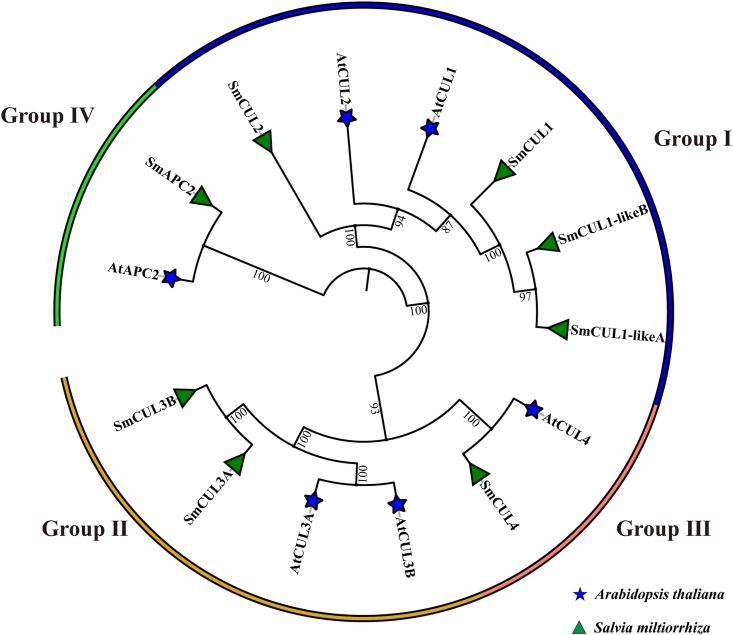
**Phylogenetic tree of *S. miltiorrhiza CUL* ubiquitin ligase genes. The phylogenetic tree was constructed by the neighbor-joining method with 1000 bootstraps.** The numbers on the nodes indicate the bootstrap values after 1000 replicates. *S. miltiorrhiza CUL* genes were clustered into four subgroups and named Group I-IV. The blue star and green triangle represented the CUL proteins in *A. thaliana* and *S. miltiorrhiza*, respectively. (For interpretation of the references to color in this figure legend, the reader is referred to the Web version of this article.)

To investigate the evolutionary history of *CUL* genes in *S. miltiorrhiza*, we constructed a phylogenetic tree using the MEGA 6.0 tool based on the CUL proteins from *S. miltiorrhiza* (8 members), *Arabidopsis* (6 members) (Fig. 1). According to the domains related to the function of CUL proteins, these specific proteins linked to CUL were classified as CUL-SCF (S-phase kinase-associated PROTEIN 1 (SKP1) -CUL-F-box), CUL-BTB (Bric a BRAC, Tramtrack and Broad Complex), CUL-DDB1 (UV-damaged DNA Binding Protein 1) and CUL-APC/C (Anaphase Promoting Complex), respectively, which were divided into four groups: CUL-SCF (Group I), CUL-BTB (Group II), CUL-DDB1 (Group III) and CUL-APC/C (Group IV). Interestingly, the total number of *CUL* genes in *S. miltiorrhiza* and *Arabidopsis* is comparatively secure, and it indicates the conservative features of this gene family.

In order to evaluate the degree of gene expansion or loss during evolution, the *CUL* genes in each group were counted. In *S. miltiorrhiza*, it was found that Groups I–IV contained 4, 2, 1, 1 *CUL* genes, respectively. In *Arabidopsis*, Groups I to IV contained 2, 2, 1, 1 *CUL* genes, respectively. Comparison of *S. miltiorrhiza* and *Arabidopsis,* the increased gene number in group I implies the presence of gene expansion in *S. miltiorrhiza.*

### 3.3 Gene structure and motif analysis of CUL genes

**Fig. 2:**
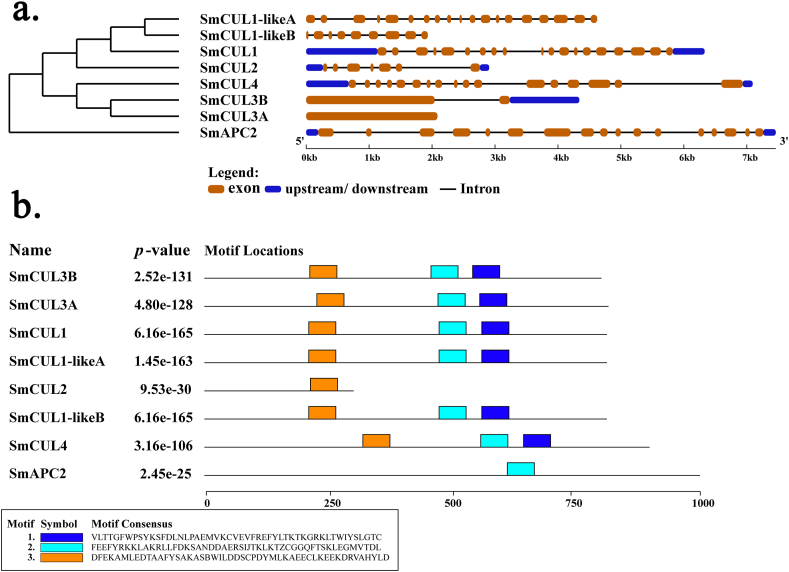
The conserved motifs and gene structure analysis of *CUL* genes in *S. miltiorrhiza*. (a) The exon/intron distribution of the eight *S. miltiorrhiza CUL* ubiquitin ligase genes was determined using the GSDS tool by comparing the coding sequences (CDS) with the relative genomic sequences. The orange box represents the CDS; the solid black line depicts the intron region and the blue box shows upstream/downstream regions. (b) Schematic representation of three motifs discovered in *S. miltiorrhiza CUL* ubiquitin ligase genes through MEME tool denoted by different colors. (For interpretation of the references to color in this figure legend, the reader is referred to the Web version of this article.)

To study the structure of *CUL* genes, we compared the full-length cDNA sequences of all genes with the corresponding genomic DNA. By comparing the number and location of exons and introns (Fig. 2a), we found that 8 *CUL* genes identified from *S. miltiorrhiza* had different numbers of exons, ranging from 1 to 19. *SmCUL1* and *SmCul1-likeA* had the largest number of exons, and all the 8 *CUL* genes contained CUL domains and about 12.5% of them had no introns. The difference in the number of exons may indicate that the *CUL* gene families have different functions involved in the secondary metabolites biosynthesis, growth and development in *S. miltiorrhiza*.

All of the identified *CUL* genes were analyzed for the presence of the novel and uncapped motifs using MEME suite utilizing a two-component finite mixture model (Fig. 2b). It was found that there are 1–3 motifs distributed in *CUL* members (Fig. S1). This discovery provides a novel evidence for predicting gene biological functions. The common motifs among the gene sequences are indicative of conserved evolutionary relatedness and similar cellular functions.

### 3.4 Cis-acting elements in the promoters of CUL genes

**Fig. 3:**
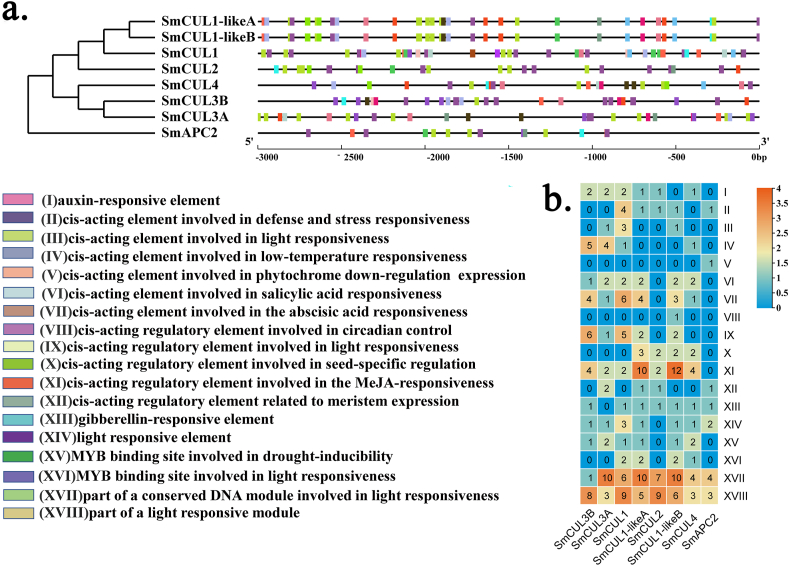
The *cis*-acting elements analysis of putative promoter of eight *CUL* genes. (a) Eighteen *cis*-acting elements includes responsive elements involved in (Ⅰ) auxin-responsive element; (Ⅱ) defense and stress responsiveness; (Ⅲ) *cis*-acting element involved in light responsiveness; (Ⅳ) low-temperature responsiveness; (Ⅴ) phytochrome down-regulation expression; (Ⅵ) salicylic acid responsiveness; (Ⅶ) abscisic acid responsiveness; (Ⅷ) circadian control; (Ⅸ) light responsiveness; (Ⅹ) seed-specific regulation; (Ⅺ) MeJA-responsiveness; (Ⅻ) meristem expression; (XIII) gibberellin response; (XIV) light response; (XV) MYB binding site related to drought-inducibility; (XVI) MYB binding site related to light responsiveness; (XVII) part of a conserved DNA module related to light responsiveness; (XVIII) part of a light responsive module. (b) The number of eighteen *cis*-acting elements of putative promoter of *CUL* genes. The color scale at the top right indicated the number of *cis*-acting elements. (For interpretation of the references to color in this figure legend, the reader is referred to the Web version of this article.)

Usually, transcription factors regulate the expression level of target genes by binding to its *cis*-acting element in the promoter of target genes in specific biological processes. Thus, *cis*-acting elements were important clues for predicting the gene function. In order to further explore the function of the *SmCUL* gene, the PlantCare database was introduced to predict the *cis*-acting elements in the putative promoter region of the *SmCUL* genes. According to the predicted data, 18 *cis*-acting elements related to stress, hormones, plant growth and development in the promoters of the eight *CUL* genes were annotated and selected to further study the distribution pattern in the promoter. As shown in Fig. 3a, different distribution patterns were observed in the promoter region of the *SmCUL* genes, indicating that they have different biological functions. Especially, the *cis-*acting elements related to hormone regulation, such as abscisic acid (ABA), salicylic acid (SA), gibberellin (GA), auxin and methyl jasmonate (MeJA) are very important in most of the *CUL* genes (Fig. 3a and b). ABRE, as a key *cis*-acting element in response to ABA treatment, has been identified in 6 *SmCUL* genes (including *SmCUL1*, *SmCUL1-likeA*, *SmCUL1-likeB*, *SmCUL3A*, *SmCUL3B* and *SmCUL4*), which indicates that most of the *CUL* genes in *S. miltiorrhiza* may be particularly sensitive to ABA treatment. In addition, 7 *SmCUL* genes (including *SmCUL1*, *SmCUL2*, *SmCUL1-likeA*, *SmCUL1-likeB*, *SmCUL3A*, *SmCUL3B* and *SmCUL4*) are confirmed to have MeJA responsive elements, and 6 *SmCUL* genes (including *SmCUL1*, *SmCUL1-likeA*, *SmCUL1-likeB*, *SmCUL3A*, *SmCUL3B* and *SmCUL4*) have *cis*-acting elements related to drought, indicating that these genes may have special resistance under drought stress. It is worth noting that the MYB transcription factor (TF) binding elements exists in the six *SmCUL* genes (including *SmCUL1*, *SmCUL1-likeA*, *SmCUL1-likeB*, *SmCUL3A*, *SmCUL3B* and *SmCUL4*), indicating that the six *SmCUL* genes may be regulated by *MYB* genes in response to drought stress. The promoter elements are clustered and represented by a word cloud image. As shown in Fig. S2, these *cis*-acting elements including light responsive element (ATTAAT), abscisic acid (ABA) responsive element (ACGTG), MeJA responsive element (TGACG and CGTCA), low temperature responsive element (CCGAAA), MYB drought-induced binding site (CAACTG), auxin responsive element (AACGAC), salicylic acid responsive element (CCATCTTTTT), gibberellin responsive element (CCTTTTG and TCTGTTG) and stress responsive element (ATTCTCTAAC), are abundant in the promoters of *CUL* ubiquitin ligase genes (Fig. 3a and b), among of them, light, ABA and MeJA responsive element **g**ot the highest abundance, implying that the *CUL* genes are might be closely related to plant growth and development.

### 3.5 Expression pattern of CUL genes exposed to ABA induction

**Fig. 4:**
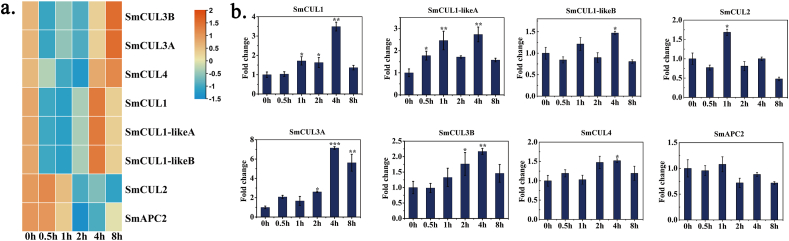
The expression profiles of eight *CUL* genes under the treatment of ABA. (a) The heat map of *CUL* genes in *S. miltiorrhiza* under ABA treatment based on RNA-seq. Genes with similar expression patterns were clustered into the same group according to the hierarchical clustering method. The top color scale indicated the Fragments Per Kilobases per Million reads (FPKM) values of each gene. (b) The expression level of eight *CUL* genes under the ABA treatment. The relative gene expression level changes were normalized to the control without ABA induction at 0h. (For interpretation of the references to color in this figure legend, the reader is referred to the Web version of this article.)

ABA has been validated to act as an abiotic inducer promoting tanshinone and phenolic acid biosynthesis in *S. miltiorrhiza* (Li et al., 2018; Deng et al., 2020b). Coincidentally, the promoters of *CUL* genes also have a large number of ABA *cis*-acting elements. So, we collected six ABA-treated RNA-Seq samples to study the expression pattern of the *CUL* genes through RNA sequencing analysis. The result showed that the identified eight *CUL* genes were all responded to ABA stress **(**Fig. 4a and Table S3**)**. Meanwhile, according to our real-time PCR results, *SmCUL1*, *SmCUL1-likeA*, *SmCUL3A* and *SmCUL3B* were significantly induced by ABA, and their expression levels peaked at 4 h (Fig. 4b). These results suggested that *CUL* genes might play an important role in ABA regulation activity.

### 3.6 Differential expression of CUL genes in various tissues

**Fig. 5:**
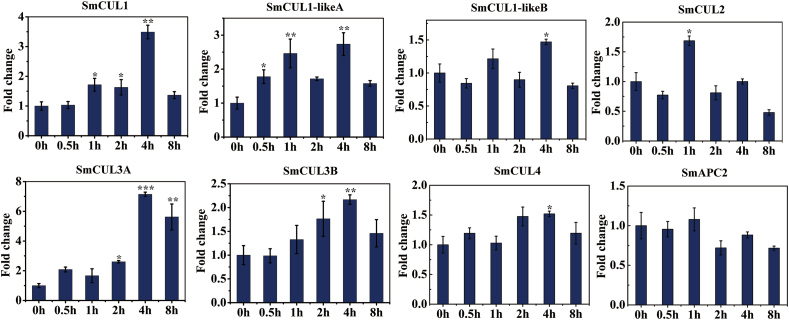
The expression profiles of eight *CUL* genes in different vegetative and reproductive tissues. Asterisks indicate significant differences in the root.

The expression profiles of the eight *CUL* genes in vegetative and reproductive tissues (leaf, stem, root and flower) were examined to explore the function of the gene participating in plant growth, development and secondary metabolism in *S. miltiorrhiza* (Fig. 5). The results showed that the expression levels of six *CUL* genes (including *SmCUL1*, *SmCUL1-likeA*, *SmCUL1-likeB*, *SmCUL2*, *SmCUL4* and *SmAPC2*) exhibited the highest expression level in the vegetative tissue of stem. Whereas, *SmCUL3A* got the highest expression level in root, which is used as the medicinal harvesting tissue in traditional chinese medicine. The differential expression of *CUL* genes in various tissues indicated the diverse function in plant secondary metabolite synthesis, growth and development in *S. miltiorrhiza*.

### 3.7 Co-expression network of CUL genes with phenolic acids and tanshinone biosynthetic genes

**Fig. 6:**
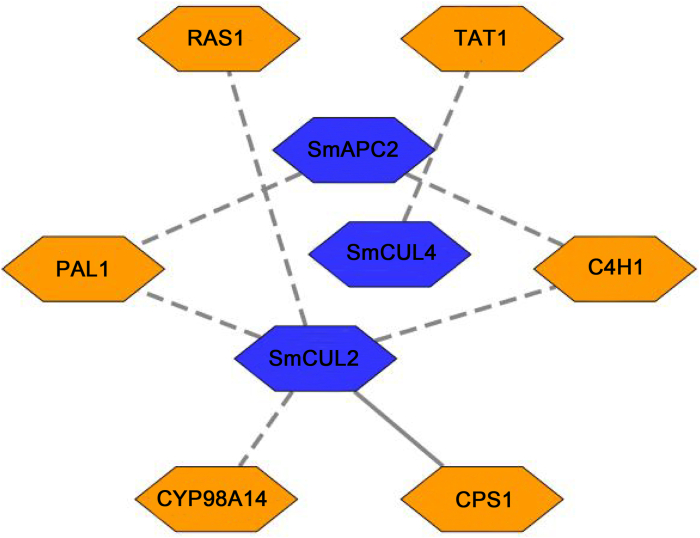
The co-expression network of *CUL* gene with phenolic acid and tanshinone biosynthetic genes. PAL1, Phenylalanine ammonia-lyase I; TAT1, Cinnamate 4-Hydroxylase I; RAS1, Rosmarinic acid synthase I; C4H1, Cinnamate 4-Hydroxylase I; CYP98A14, Cytochrome P450 family 98A subfamily oxidase 14.

Previous studies showed that ABA could promote the phenolic acids and tanshinone accumulation in hairy roots by activating the expression of phenylalanine ammonia-lyase (PAL), tyrosineamino transferase (TAT) (Zhang et al., 2013; Ding et al., 2017; Deng et al., 2020b; Zhou et al., 2021b).The co-expression network of eight *CUL* genes with phenolic acids and tanshinone biosynthetic genes was constructed, and the result indicated that 3 out of 8 *CUL* genes (including *SmAPC2*, *SmCUL2* and *SmCUL4*) showed a negative correlation with phenolic acids biosynthetic genes (Fig. 6 and Table S4). Co-expression analysis revealed that 1 *CUL* gene (*SmCUL2*) showed a positive correlation with *CPS* gene in tanshinone biosynthetic pathway with Pearson correlation coefficient(r) > 0.8 and *P*-value <0.05 as a cutoff (Fig. 6 and Table S4). Overall, these results suggested that 3 out of 8 *CUL* genes might participate in phenolic acids and tanshinone biosynthesis.

## 4 Discussion

*CUL* ubiquitin ligase genes widely exist in plants and have been validated to participate in diverse biological functions (Adams and Spoel 2018; Ma et al., 2021; Yu et al., 2021). Due to *CUL* genes acting as an essential role during plant development, they have been identified in many plant species. *S. miltiorrhiza*, one species from *Lamiaceae*, is a valuable traditional Chinese herbal plant being historically used to treat cardiovascular and cerebrovascular diseases (Deng et al., 2020a; Zhou et al., 2021a). Genome-wide identification of the *CUL* genes is an essential process towards further functional characterization of these genes in *S. miltiorrhiza*, but this work is poorly studied till now. In this study, 8 *CUL* genes were identified in *S. miltiorrhiza* genome by HMMER analysis using the Pfam and InterPro databases, and the total number of *CUL* genes in *S. miltiorrhiza* is comparable to that of *A. thalania* (11) (Thomann et al., 2005; Liu et al., 2017).

In *Arabidopsis*, 6 out of 11 *CUL* genes have complete C-terminal or N-terminal sequence, and the other five *CUL* genes cannot be translated normally because they do not have complete N-terminal and C-terminal sequence. Based on the results of evolutionary tree clustering, eight *CUL* genes in *S. miltiorrhiza* were named according to the names of *CUL* genes in *A. thaliana* (Fig. 1). Phylogenetic analysis showed that a total of 14 CUL protein members in these two species (8 in *S. miltiorrhiza* while 6 in *A. thalania*), were grouped into four groups (Fig. 1) (Ban and Estelle 2021). Except to the CUL domain, these members of SmCUL1, SmCUL1-likeA, SmCUL1-likeB, SmCUL3A, SmCUL3B, SmCUL4 and SmAPC2 also contain Cullin_Nedd8, APC2 or ANAPC2 domains (Zhuang et al., 2009).

In this study, the CUL proteins can be divided into four groups, including CUL-SCF (Group I), Cul-BTB (Group II), Cul-DDB1 (Group III) and Cul-APC/C (Group IV). CUL ubiquitin ligases can form multisubunit enzymes with complex structures (Thomann et al., 2005). The interaction of multisubunit enzymes with substrates requires specific connexin to form specific complexes in order to act as E3 ubiquitin ligases properly, of which it indicates the functional complexity and diversity of the *CUL* gene family (Chen et al., 2015; Liu et al., 2017; Chico et al., 2020).The diverse structure and organization of the *CUL* genes, is associated with the evolution and functional differentiation of this gene family in different species (Wu and Krainer 1998; Thomann et al., 2005). In the present study, some *CUL* genes in *S. miltiorrhiza* either have no introns or more than three introns (Fig. 2). It was thought that a large number of introns in *CUL* genes might act as a mutational buffer to protect the coding sequence and keep away from functionally deficient mutations (Wu and Krainer 1998; Thomann et al., 2005). The results of gene structure and motif analysis of *CUL* genes in *S. miltiorrhiza* will be valuable for predicting the gene evolution and identifying the function of candidate genes.

Through analyzing *cis*-acting elements within promoters, it indicates that the *CUL* gene family is involved in stress-related mechanisms, hormone regulation, growth and development (Fig. 3A) (Belda-Palazon et al., 2019; Chen et al., 2020b; Dou et al., 2021). In our study, most of *CUL* genes contained ABA responsive elements in putative promoter regions (Fig. 3B). In particular, six of the eight *CUL* gene (*SmCUL4*, *SmCUL3A*, *SmCUL3B*, *SmCUL1-likeA*, *SmCUL1-likeB* and *SmCUL1*) promoters all contained ABA responsive elements, among of which *SmCUL1* contains the largest number of ABA responsive elements reaching to six. The result indicates that the *CUL* gene may play an essential role in the ABA signal transduction process in *S. miltiorrhiza*. In *A. thaliana*, AtCUL3 was validated to interact with AtHB6 to respond to ABA induction (Lechner et al., 2011). Meanwhile, AtCUL3 promoted the degradation of AtMYB56 and AtWRI1 to regulate fatty acid accumulation in seeds and to affect flowering (Chen et al. 2013, 2015; Škiljaica et al., 2019). Herein, many MYB transcription factors binding sites referred to drought induction within the promoter region of *CUL* genes (including *SmCUL1*, *SmCUL1-likeA*, *SmCUL1-likeB*, *SmCUL3A*, *SmCUL3B* and *SmCUL4*) were identified in *S. miltiorrhiza*, suggesting that *CUL* genes might be regulated by related *MYB* genes mediating the drought stress signaling (Park et al., 2008; Baldoni et al., 2015; Chen et al., 2015), and this hypothesis needs to verify by further experiments. We also found light responsiveness elements, low-temperature responsive elements, and gibberellin-responsive elements in the promoter regions of *CUL* genes in *S. miltiorrhiza*. These results indicated that *CUL* genes might participate in diverse biological processes during growth and development in *S. miltiorrhiza* (Roberts et al., 2011; Morimoto et al., 2017).

The *CUL*genes were thought to participate in various abiotic stress and hormone induction (Zhang et al., 2014; Orosa et al., 2017). Due to the highest occurrence frequency of the ABA responsive elements in the promoters of *CUL* genes (Fig. 3), it pushed us to investigate the gene expression pattern of the *CUL* genes responding to ABA treatment. Based on RNA-sequencing databases, it revealed that except to SmAPC2, the other seven *CUL* genes could be induced by ABA treatment (Fig. 4). Our quantitative detection of the expression level of all *CUL* genes exposed to ABA treatment were consistent with the transcriptome database (Fig. 4b) In fact, we found that many *cis*-elements in the *CUL* gene family are associated with hormone regulation not only contain ABA, but also include SA, GA and other auxins (Fig. 3).

In conclusion, we have characterized the *CUL* gene family in *S. miltiorrhiza* based on the whole genome, transcriptome dataset and qRT-PCR expression analysis. Our research is the first systematic and comprehensive analysis of the *CUL* genes family in *S. miltiorrhiza*, and provides a valuable information for further elucidating the molecular mechanism of *CUL* genes responding to ABA induction. It may also help us to recognize the diverse biological functions of *CUL* genes in other species.

## CRediT authorship contribution statement

**Xiankui Gao:** Writing – original draft, Drafting the manuscript, Validation, Methodology, Formal analysis, Funding acquisition, of, Data curation. **Xiujuan Li:** Writing – original draft, Drafting the manuscript, Formal analysis, Funding acquisition, of, Data curation. **Chengan Chen:** Formal analysis. **Can Wang:** Formal analysis. **Yuqi Fu:** Resources, Investigation. **ZiZhen Zheng:** Resources, Investigation. **Min Shi:** Writing – review & editing, Supervision. **Xiaolong Hao:** Writing – review & editing. **Limei Zhao:** Methodology. **Minghua Qiu:** Writing – review & editing. **Guoyin Kai:** Conceptualization, and design of study, Acquisition of data, Revising the manuscript. **Wei Zhou:** Writing – original draft, Drafting the manuscript, Formal analysis, Conceptualization, and design of study, Acquisition of data, Revising the manuscript, Approval of the version of the manuscript to be published.

## Declaration of competing interest

The authors declare that they have no known competing financial interests or personal relationships that could have appeared to influence the work reported in this paper.
