# Supplementary material for: Mining of the CULLIN E3 ubiquitin ligase genes in the whole genome of Salvia miltiorrhiza
Source: Curr Res Food Sci. 2022 Oct 8;5:1760–8. doi: 10.1016/j.crfs.2022.10.011 (PMC9576582; doi:10.1016/j.crfs.2022.10.011)
Supplement: Multimedia component 1 [file mmc1.doc]

**Supplementary Figures**

**Fig. S1.** Three motifs identified using MEME suit for *CUL* gene family.

**Fig. S2.** The word cloud image of *cis*-acting elements in the promoter of 8 *CUL* genes.

| **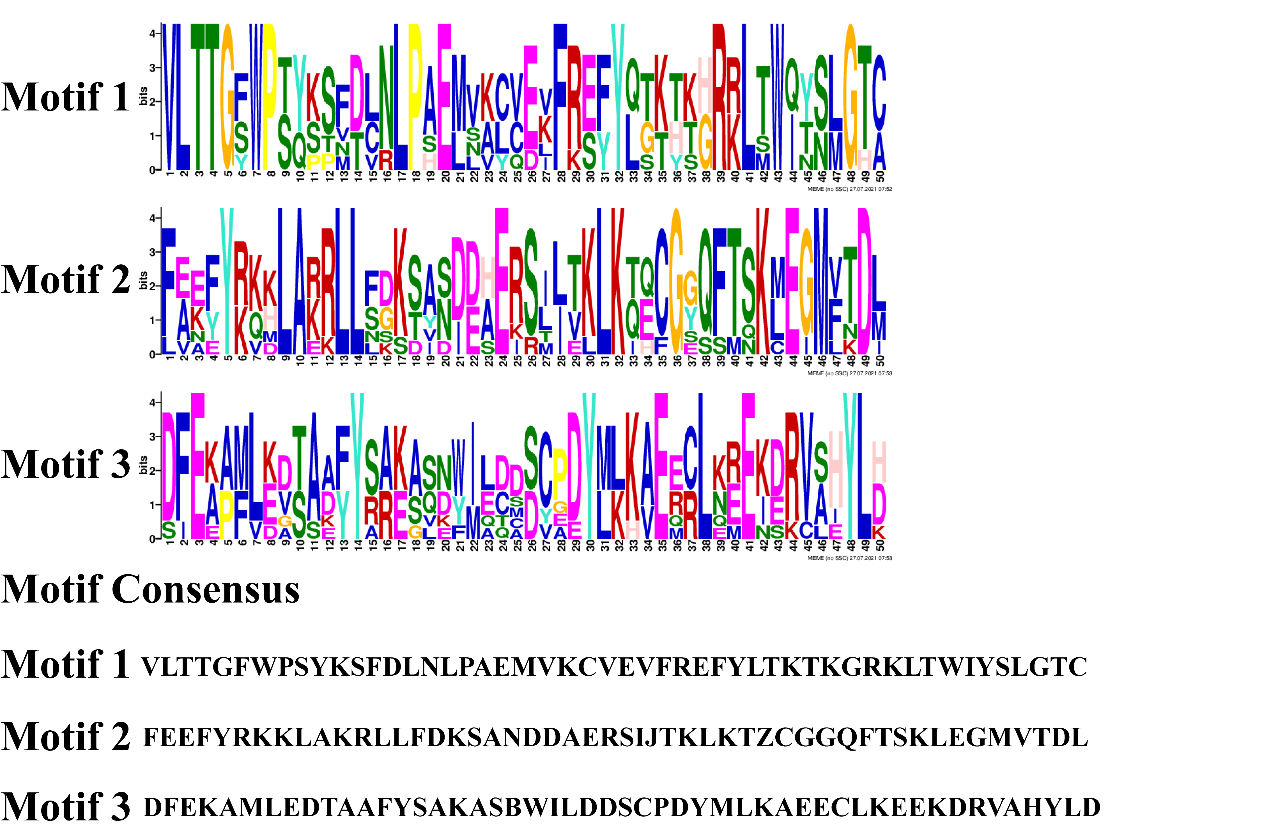** |
| --- |

**Fig. S1**

| **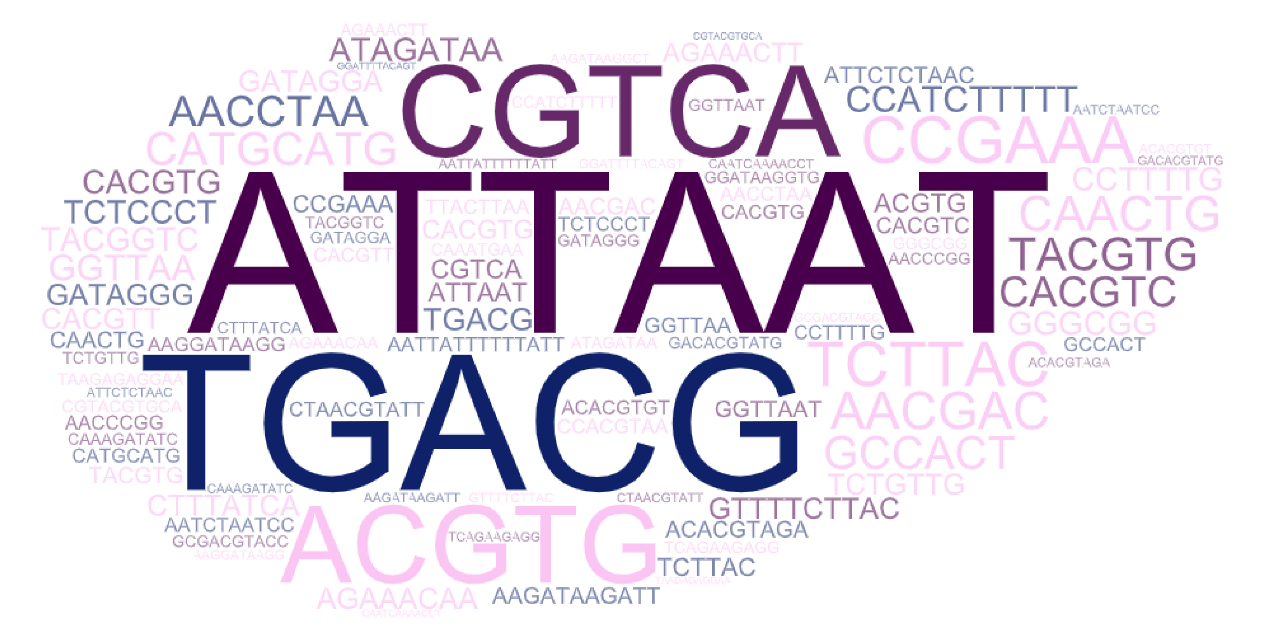** |
| --- |

**Fig. S2**
